# Characterization by Spectroscopic and Microscopic Techniques of Degraded Zinc White Pigment in *Las Dos Fridas*

**DOI:** 10.1021/acsomega.4c10530

**Published:** 2025-04-18

**Authors:** Pablo Aguilar-Rodríguez, Sandra Zetina, Adrián Mejía-González, Nuria Esturau-Escofet

**Affiliations:** † Instituto de Química, Universidad Nacional Autónoma de México, México City 04510, México; ‡ Instituto de Investigaciones Estéticas, Universidad Nacional Autónoma de México, México City 04510, México

## Abstract

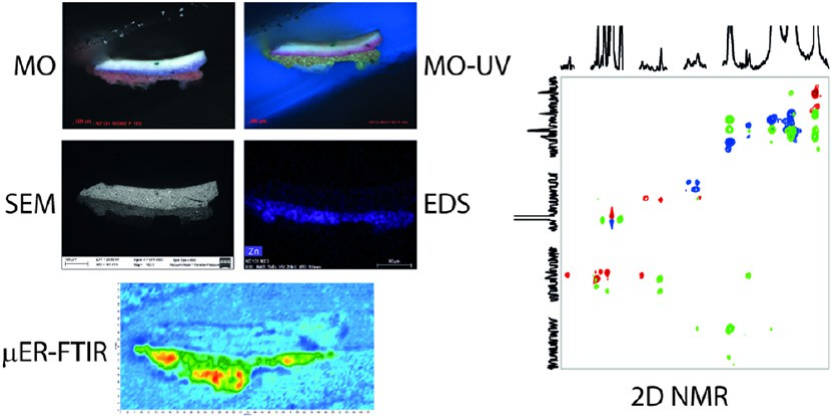

*Las Dos Fridas*, 1939, is Frida Kahlo's most renowned achievement. This work now presents conservation problems, especially around white regions. In 2008, ten microsamples of *Las Dos Fridas* were studied by scanning electron microscopy with energy-dispersive X-ray spectroscopy. Recently, to understand its alterations, the microsamples were reanalyzed using nuclear magnetic resonance spectroscopy, micro-Fourier transform infrared spectroscopy, attenuated total reflection infrared spectroscopy, and electron microscopy. The analysis revealed a pictorial layer affected by metallic soaps and the presence of zinc lactate, which is typically associated with environmental contamination. A metal-catalyzed radical pathway is posited by this study. According to this hypothesis, the oxidative drying processes of linseed oil generate free radicals that degrade the cellulose of the cotton canvas. This hypothesis is based on the pictorial technique employed by Frida Kahlo, whereby the absence of a preparation base for her canvas is highlighted, which resulted in the transmission of the drying oil to the canvas fabric.

## Introduction

**Figure 1:**
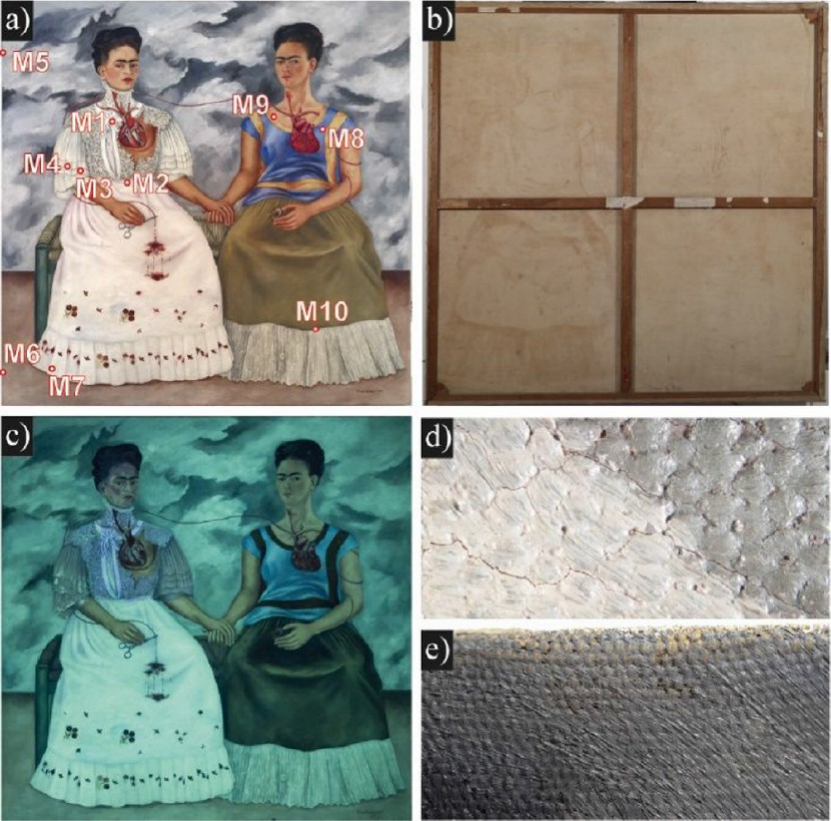
(a) *Las Dos Fridas*, by Frida Kahlo, 1939, oil on cotton canvas, 173.5 cm × 173.5 cm. *Museo de Arte Moderno*. Photo: Pedro Ángeles, LANCIC, IIE, UNAM. Sampling at points M1–M10 are indicated. All rights reserved: copyright 2024 *Banco de México*, *Fiduciario en el Fideicomiso relativo a los Museos* Diego Rivera y Frida Kahlo. *Av. Five de Mayo No. 2, col. Centro, alc. Cuauhtémoc, c.p. 06000, Ciudad de México. Reproducción autorizada por el Instituto Nacional de Bellas Artes y Literatura* (INBAL). (b) Reverse of *Las Dos Fridas*, (c) *Las Dos Fridas* under UV light (Hand-held UVP UV Lamp 365 nm), Pedro ngeles, LANCIC IIE, UNAM, (d) close-up of the white skirt area, and (e) close-up to edge of canvas.

The double self-portrait *Las Dos Fridas* (Figure 1a) is possibly Frida Kahlo's (1907–1954) most celebrated canvas. Painted in 1939 alongside the now lost *La Mesa Herida* for the International Surrealist Exhibition, the painting was acquired in 1948 to form the founding collection of the Museum of Modern Art from the National Institute of Fine Arts in Mexico City (*Museo de Arte Moderno,* from the *Instituto Nacional de Bellas Artes y Literatura*, MAM-INBAL).

*Las Dos Fridas* marked a significant departure from Kahlo’s previous work, as it was her first large-format canvas. This shift from her usual practice of creating small altarpiece paintings represents a considerable change in her artistic approach. Kahlo used a medium-weave cotton canvas (Figure S1). The dimensions of the work are unusually squared 173.5 cm × 173.5 cm, matching the width of the textile roll. This suggests that the artist might have stretched a commercial cotton canvas on the very light wooden frame with movable joints, box, and dowel assemblies of six members, with interior cross braces (Figure 1b).

Frida Kahlo worked at a rapid pace to create *Las Dos Fridas*, and there are many photographs that show her painting it. This work is one of the most widely reproduced works of the National Mexican artistic heritage. In 2008, conservators from the National Center of Conservation and Registrar of Movable Artistic Heritage (*Centro Nacional de Conservación y Registro del Patrimonio Artístico Mueble*, CENCROPAM, INBAL) identified several alterations that could impede its frequent transportation. Conservators documented varying degrees of deterioration across different regions of the painting, characterized by pronounced fine cracking and localized areas of paint loss and surface erosion. These degradations were particularly evident in the white skirt and other white regions (Figure 1d). In 2008, CENCROPAM asked LDOA, IIE, and UNAM to study *Las Dos Fridas* to understand its degradation.^1^

During the 19th and 20th centuries, zinc white pigment was widely used. Zinc oxide represents a significant advancement in the field of paint technology, offering a safer alternative to lead white pigment. However, the reactivity of zinc oxide in oil-based media presented several challenges, including the formation of zinc soaps, which has been linked to the deterioration of paintings, such as chalking, cracking, delamination, and paint loss. These issues can significantly alter the appearance of the artwork.^1−3^

The use of zinc oxide as an undercoat in easel paintings has been associated with the formation of brittle films that are prone to cracking.^4^ Osmond et al. performed a cross-sectional analysis of deteriorated easel paintings with zinc grounds using synchrotron Fourier transform infrared microspectroscopy (SR-μFTIR).^5,6^ The analysis identified the presence of not only metallic zinc soaps but also zinc lactate, which is typically attributed to environmental factors.^5,7−10^

Pratali conducted a study in which various mockups of zinc white and other white pigments were applied on glass or linen canvas and subjected to artificial weathering. The study revealed that only the mixture of zinc white with poppy or linseed oil on linen canvas exhibited cracking, indicating a possible correlation between crack formation and reactivity of the support.^11^

The alterations observed in *Las Dos Fridas* can likely be attributed to a series of factors, including the experimental approaches to the painting’s format, material composition, and the techniques employed by Kahlo. Through the study of microsamples, the degradation patterns in the artwork, combined with an analysis of its painting materials and technique and current conservation state, we propose an alternative mechanism for the formation of zinc lactate from zinc white. This study is based on a new assessment, with attenuated total reflectance-Fourier transform infrared spectroscopy (ATR-FTIR), nuclear magnetic resonance (NMR) spectroscopy, external reflection-Fourier transform infrared microscopy (μER-FTIR), optical microscopy (OM), and scanning electron microscopy with energy dispersive spectroscopy (SEM -EDS), of the microsamples obtained in 2008.

## Materials and Methods

### In Situ Analysis Methods

In 2008 prior to sampling, *Las Dos Fridas* was analyzed and documented through infrared (750 nm filter, 600 W tungsten lamp), visible, and ultraviolet (Hand-held multiband UVP UV Lamp 365 nm) imaging performed with a Phase One Mamiya RBZ Pro II camera and microphotographs of the surface by LDOA IIE, UNAM.

### Microscopic Cross-Sectional Analysis

LDOA, IIE, and UNAM provided ten microsamples from the easel painting obtained in 2008. These microsamples were taken from the painting and divided using a surgical scalpel. The microscopical fragments were mounted as cross-sections employing the methacrylic resin Claro Cit (Roper Technologies, Sarasota, FL, USA). The resin-embedded microsamples were sanded to expose the paint stratigraphy and polished using SiC grinding papers (800–4000 grit).

The sampling criteria followed the observation of UV photography and surface microscopy studies in which different white paintings were detected; to understand the composition and changes of those regions, six of the ten microsamples were from white areas. The other four microsamples were also taken to study changes in composition shown through IR studies.

Microsamples prepared as cross-sections were studied in 2024 with an Axio Imager Z2 optical microscope (Carl Zeiss, Oberkochen, Germany) equipped with a Xenon arc lamp for UV fluorescence with 430–465 and 465–500 nm filters and a HAL100 light source in reflected light mode.

Morphological and elemental analyses were performed using an EVOMA25 SEM (Carl Zeiss, Oberkochen, Germany) equipped with a 30 mm EDS microprobe (Bruker, Bremen, Germany). Double-stick carbon tape was used to attach and render the microsamples conductive. BSE imaging was performed at variable pressure (80 Pa) under nitrogen flow. EDS spectra and maps of characteristic X-ray photon emission energies from the microsamples were acquired using 15.0–20.0 kV accelerating voltage.

Infrared maps of the cross section were acquired with an Agilent Cary 670 FTIR spectrometer interfaced to an Agilent Cary 620 FTIR microscope equipped with a 64-pixel focal plane array detector that comprises a two-dimensional array of detector elements. Data were collected in reflection mode over a spectral range of 4000–900 cm^–1^ with 4 cm^–1^ spectral resolution by coadding 100 scans per point and processed using Agilent Resolutions Pro v. 5.0 software (Agilent Technologies, Santa Clara, CA, USA).

### Spectroscopic Analysis of Microsamples

Four of the ten microsamples slivers were analyzed by ATR-FTIR spectroscopy and then dissolved in deuterated chloroform (CDCl_3_) for NMR analysis.

ATR-FTIR was performed with a Cary 600 spectrophotometer (Agilent Technologies, Santan Clara, CA, USA). Data were collected in absorbance mode in a spectral range of 4000–400 cm^–1^ with 128 scans at 4 cm^–1^ spectral resolution. All data were processed with SpectraGryph v. 1.2.16.1 (developed by Friedrich Menges Am Dummelsmoos 28, 87561 Oberstdorf, Germany) and the ELViS module of MestReNova (v. 14.3.3, Mestrelab Research SL, Santiago de Compostela, Spain) software. The signal identification was compared to that in the published literature.

NMR spectra were acquired with a Bruker Avance III HD 700 spectrometer (Bruker, Billerica, MA, USA) operating at 16.4 T (700 and 175 MHz for ^1^H and ^13^C frequencies, respectively) equipped with a 5 mm *z*-axis gradient TCI cryoprobe. ^1^H NMR chemical shift (δ) is reported in ppm relative to the solvent resonance as an internal standard (CDCl_3_; δ_H_ = 7.26 ppm). ^1^H NMR and two-dimensional experiments in COSY, ed-HSQC, and HMBC were acquired at 300 K with standard pulse sequences from the Bruker library and processed with MestReNova software.

## Results and Discussion

### UV-Induced Fluorescence and IR Imaging

**Figure 2:**
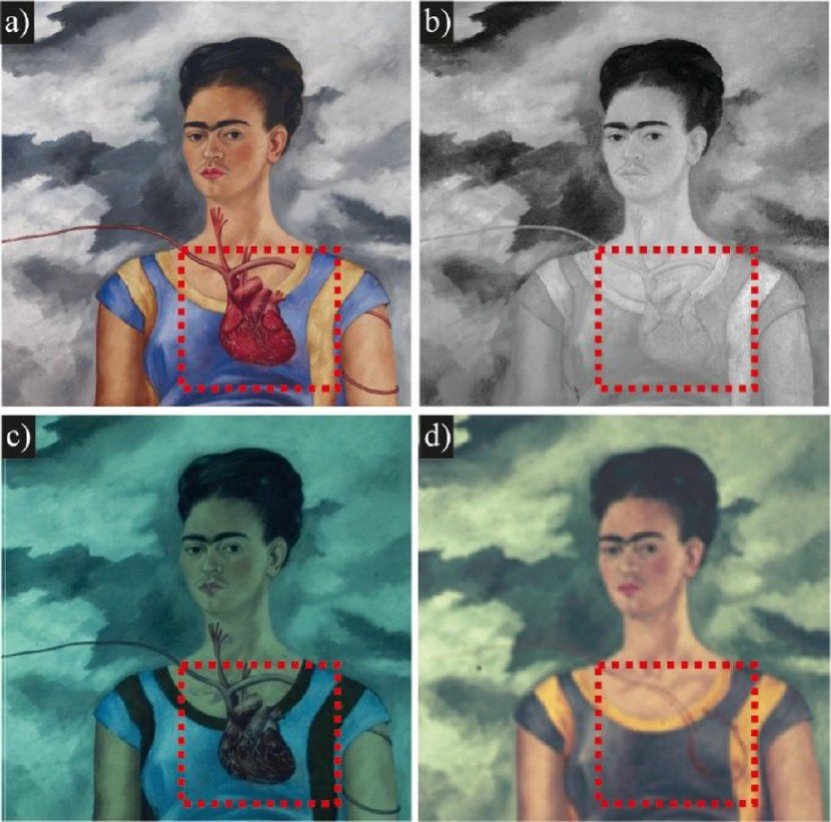
(a) Zoomed-in view of *Las Dos Fridas* in 2008 LDOA LANCIC IIE UNAM. (b) Infrared close-up photograph of the juchiteca Frida, captured using a 750-nm filter and a 600 W tungsten lamp LDOA LANCIC IIE UNAM. (c) Section of the photograph under UV light. (d) Detail of the initial placement of the heart, in Nickolas Muray ca. September 1939 photo, George Eastman House, Rochester New York. All rights reserved: copyright 2024 *Banco de México, Fiduciario en el Fideicomiso relativo a los Museos Diego Rivera y Frida Kahlo. Av. Five de Mayo No. 2, col. Centro, alc. Cuauhtémoc, c.p. 06000, Ciudad de México. Reproducción autorizada por el INBAL*.

The white regions in *Las Dos Fridas* exhibit distinct types of fluorescence under UV light (Figure 1c): the skirt acquired a greenish tone, the ruffles near the heart appeared as a blue hue, and the sleeve a darker grayish green tone. The numerous corrections show that Kahlo meticulously overpainted the composition and made several changes and adjustments with different white pigments to modify the white dress in the portrait on the left. A pentimento can also be observed in Figure 2 by comparing close-up images from infrared and ultraviolet photography along with a historical photograph taken by Nicholas Muray. In the depiction of Frida on the right, dressed as a *juchiteca*, it is evident that the heart was originally painted further to the right and finally moved to the center of the figure. In the entire area of the white skirt, extensive cracking and paint peeling are evident (Figure 1d). It seems that Kahlo modified the hue of the skirt shortly after its initial application. In the previous version, she painted a purple skirt instead of white, as can be seen in a color photograph taken in 1939 (Figure S2), resulting in the current white appearance with purple nuances. This modification led to poor adhesion between the two layers of paint. Combined with the insufficient preparation of the canvas and its natural movement over time, these factors have contributed to differential stresses and the subsequent cracking of the paint. Most of the microsamples have a lack of preparation ground, as seen in Figure 1e, where the weft of the canvas can be seen through the pictorial layer. The painting was possibly sized with a light application of hide glue and a red paint layer, as was noted only in one area in M2 (Table 1). The lack of adequate sealing or a proper preparatory base caused the textile to absorb much of the linseed oil by wicking, leading to the oil transferring to the reverse side of the canvas (Figure 1b). Early conservation reports noted this phenomenon, along with the development of craquelures.

**Table 1:**
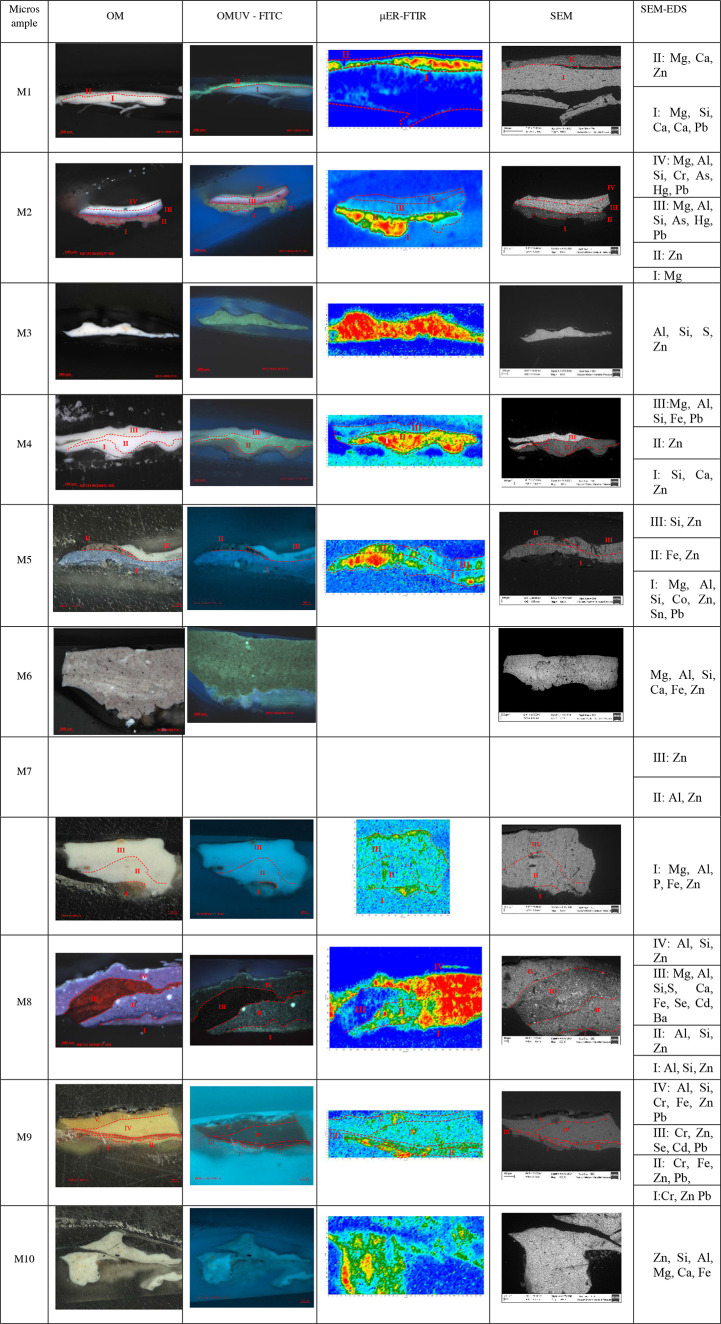
Optical Microscopy with Polarized Light UV Filter, μER-FTIR Chemical Map Displaying 1321 cm^–1^, Backscattered Electron Micrograph, and EDS Summary by Layers of Cross Section from *Las Dos Fridas*

### Paint Chemical Characterization

#### NMR

**Figure 3:**
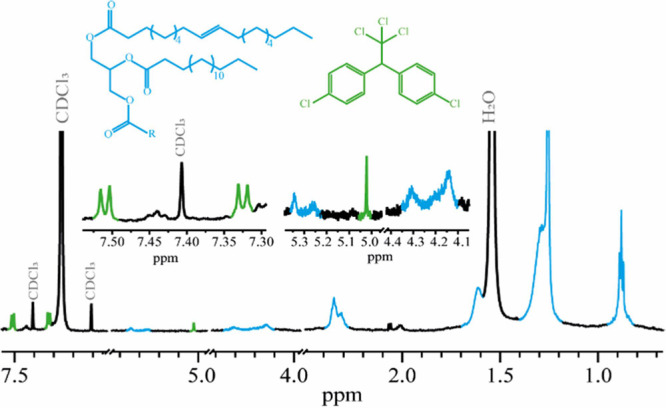
^1^H NMR spectra (700 MHz, 300 K, CDCl_3_) of M5. Signals and structure of compound identified are colored: blue triglycerides and DDT in green.

Figure 3 shows ^1^H NMR (700 MHz, 300 K, CDCl_3_) of M5. The broad signals at δ_H_ 0.88, 1.25, 1.29, 1.33, 1.62, 2.01, 2.34, 4.15, 4.29, 5.26, and 5.35 ppm correspond to the nonpolymerized oleic and palmitic triglyceride from the linseed oil. The linoleic triclyceride, or linoleic acid, was not identified, which indicates that the binder is almost completely dry. Unexpectedly, the spectrum also shows signals at δ_H_ 7.32 (4H, d, *J* = 8.6 Hz), 7.51 (4H, d, *J* = 8.4 Hz), and 5.02 (s) ppm, which correspond to dichlorodiphenyltrichloroethane (DDT). The elucidation of the identified compounds with the 1D and 2D NMR spectra is detailed in the Supporting Information (Figures S3 and S4; Tables S1 and S2).

#### SEM-EDS

The elemental composition of each layer is summarized in Table 1. The combined OM and SEM-EDS analysis revealed a very direct stratigraphy in the cross-section microsamples; most of them have between one and three layers that reflect the color on the surface. Thus, in general, Kahlo’s technical approach was very direct, except for the aforementioned changes in the color of the skirt and the location of the heart. The EDS data allow us to propose a possible color palette used by Kahlo, which will be discussed in detail subsequently. The white pigments present two different compositions; some have prominently Zn and others Pb. In most of the layers, there are noncolored particles with Al, Si, sometimes Mg, and Ca. Three different red layers were detected: (i) one containing a rich Hg red pigment (M2, layer III), (ii) another with Se, Cd, and Fe (M8, layer III) which additionally has Ba, Zn, and Pb, and (iii) the red artery in the yellow blouse or *huipil* has a red pigment with Se and Cd, and there is also Cr, Pb, and Zn (M9, layer III). The yellow layers are rich in Cr and Fe pigments and also have Al, Si, Zn, and Pb (M9, layers I, II, and IV). The gray in the clouds was obtained by mixing a Zn white pigment with Fe and possibly C (M5, layer II). The brown ground has Fe pigments with Zn, Ca, Al, Si, and Mg (M6). The blue of the sky presents Co, Sn, but also Mg, Al, and Si (M5, layer I), and were mixed with white pigments with Zn and Pb.

**Figure 4:**
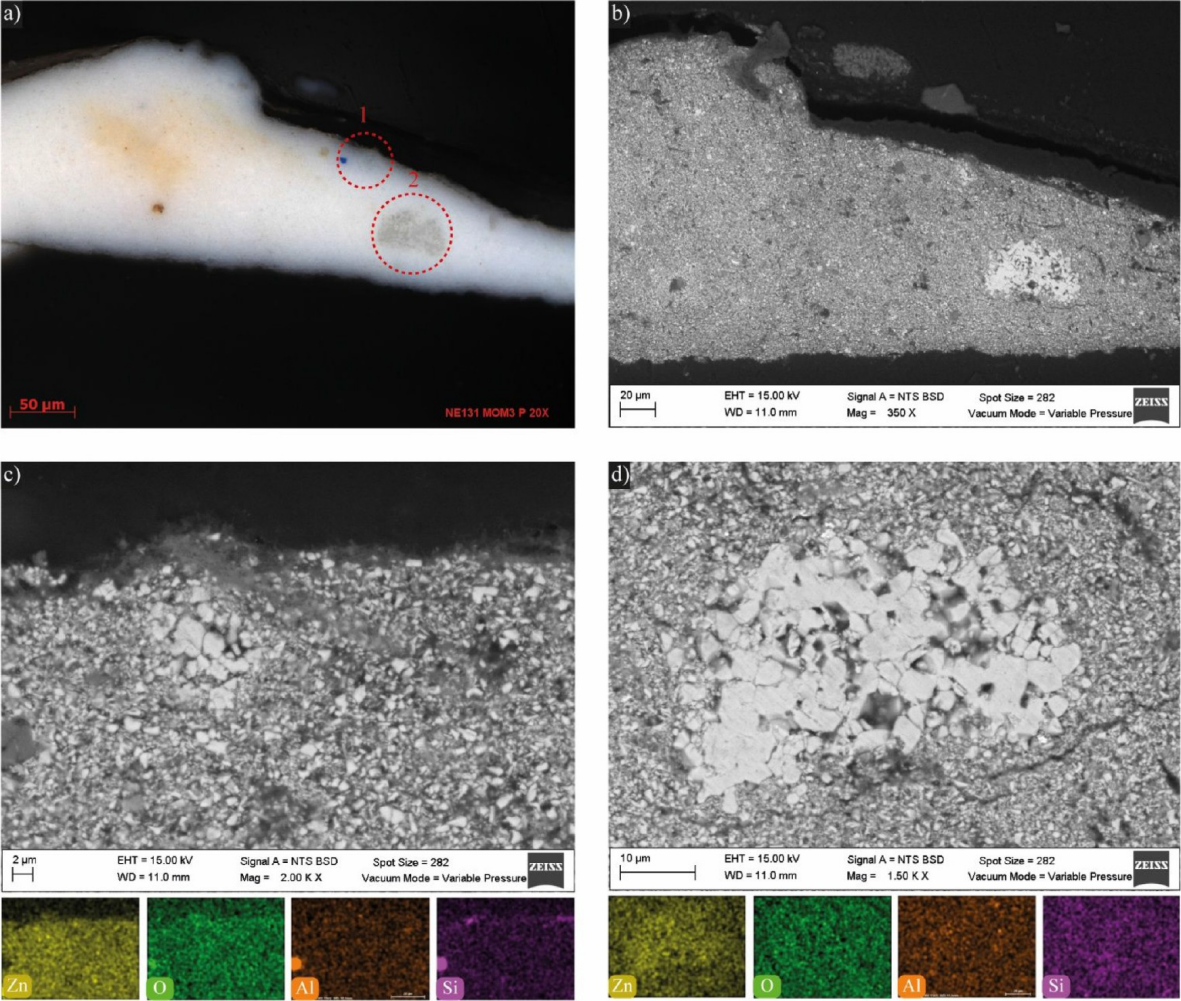
Micrographs of cross-section from M3 of *Las Dos Fridas*: (a) Polarized light OM (two regions of interest are highlighted). SEM micrographs (BSE, 15.0 kV) and elemental mappings of Zn, O, Al and Si, (b) cross-section (350×), c) close-up of region 1 (2000×), and d) close-up of region 2 (1500×).

Figure 4 shows both optical (Figure 4a) and electron photomicrographs (Figure 4b–d) of the M3 cross-section from white-dressed Frida. The optical micrograph (Figure 4a) displays two grayish areas highlighted by dotted lines. In the BSE micrograph (Figure 4b), these areas appear as conglomerates of acicular particles. Figure 4c,d provides close-up views of these conglomerates and their corresponding EDS maps, revealing the presence of Zn within these structures.

#### ATR-FTIR

**Figure 5:**
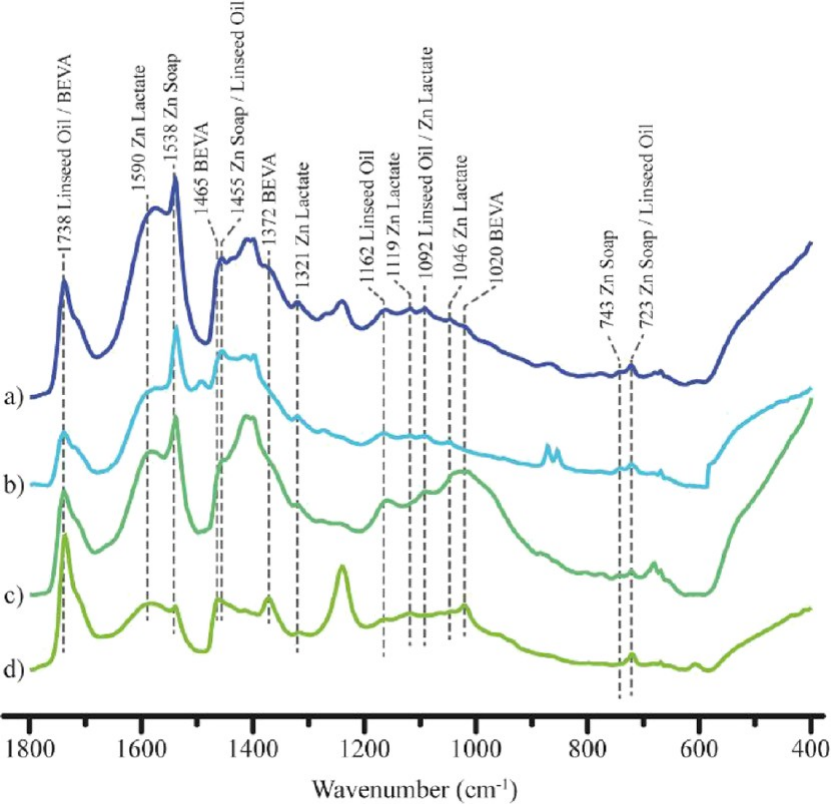
ATR-FTIR spectra from 1800 to 400 cm^–1^ with signal assignments of identified compounds in (a) M3, (b) M4, (c) M5, and (d) M7 from *Las Dos Fridas*.

Microsamples slivers M3, M4, M5, and M7 taken from white regions were analyzed with ATR-FTIR (Figure 5). The observed signals at 1738 (s, ν_s_(C=O)), 1455 (m, CH_2_, CH_3_ δ(CH_2_)), 1162 (s, ν_s_(C–O) + ν_as_(C–O)), 1092 (m, ν_s_ (C–O)), and 723 cm^–1^ (vw, cis δ (−C=C–H) + CH_2_ skeletal vibration) are attributed to linseed oil.^12−14^ The ATR-FTIR signal assignment was based on the aforementioned NMR structure elucidation. The signals at 1590 (vs, ν_as_ (COO^–^)), 1321, 1119 (ν_s_ (C–O)), 1092 (ν_s_ (C–O)), and 1046 cm^–1^ are attributed to zinc lactate.^9^ The signal at 1590 cm^–1^ could also be attributed to oxo-type ionomeric zinc carboxylates of linseed oil.^15−17^ The signals at 1538 (vs ν_as_ (COO^–^)), 1455 (s, ν_s_ (COO−)), 743, and 723 cm^–1^ are attributed to zinc soaps.^9,18−20^ The signals at 1738, 1465, 1372, and 1020 cm^–1^ are attributed to poly(ethylene-*co*-vinyl acetate) of BEVA film, a common restoration resin.^21^

## Degradation Analysis

### μER-FTIR

**Figure 6:**
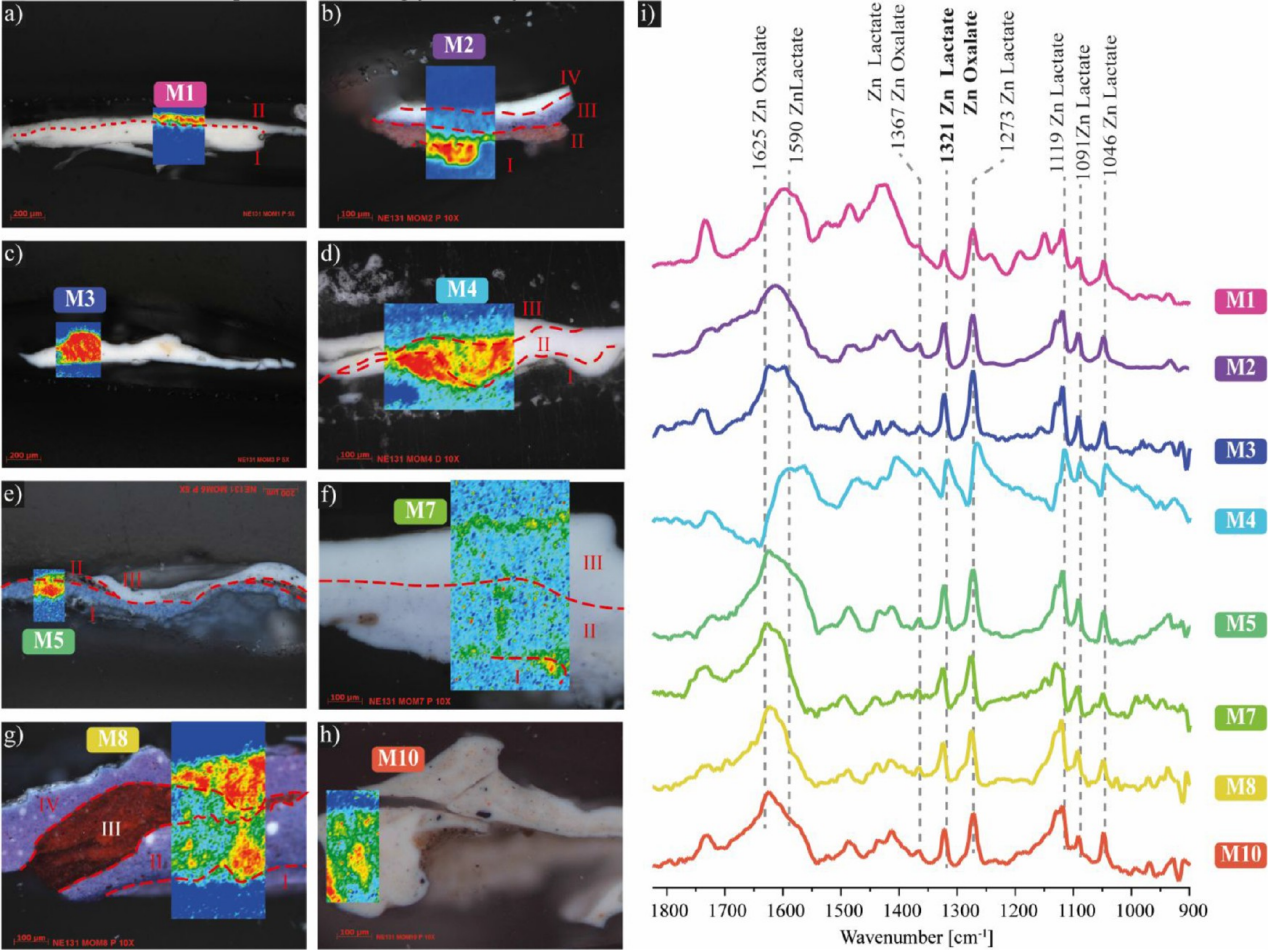
Polarized light micrograph and overlap of μER-FTIR chemical map displaying the 1321 cm^–1^ band distribution of the cross-section from (a) M1, (b) M2, (c) M3, (d) M4, (e) M7, (f) M7, (g) M8, (h) M10, and (i) μER-FTIR spectra from each chemical map indicating the bands attributed to zinc lactate and oxalate.

Figure 6 presents the μER-FTIR chemical maps as heatmaps, where color variation represents the intensity of the mapped signal (higher intensity appearing in red and lower intensity in blue). These maps depict the distribution of the 1321 cm^–1^ band, a characteristic signal of zinc lactate, within the cross-sections of the analyzed microsamples. The spectra include signals at 1367, 1321, 1273, 1119, 1091, and 1046 cm^–1^, confirming the presence of zinc lactate throughout most of the stratigraphic sequence. The signals at 1625, 1367, and 1321 cm^–1^ can also be attributed to zinc oxalate.

## Discussion

SEM–EDS analysis of *Las Dos Fridas* reveals Frida Kahlo’s color mixing and her frequent choice of using two pigments of the same color to obtain nuanced tones.

She used zinc and lead white in alternate layers; they can be found in M1, M2, M3, M4, M5, M7, and M10. Zinc white layers present an intense fluorescence in OMUV-FITC (Table 1).

It is possible that Kahlo mixed zinc white commercial oil paint with different colors since the chemical composition (Al, Si, Mg, Ca) in most layers suggests the use of aluminosilicate and calcium carbonate fillers typical of commercial paints. However, there are also some white layers that contain mainly lead white, sometimes applied as touches of light, since many have no other fillers.

To produce paler shades of colors, Kahlo alternatively used zinc and lead white, sometimes mixing the two, which is not observed in the white layers.

The painter used at least three reds: vermillion, cadmium, and iron red. Vermillion was found only in M2, the red layer under the purple, attributed to the presence of Hg and S, mixed with zinc white. The red layers that correspond to the heart and arteries in M8 and M9 were possibly prepared with cadmium red related to Cd and Se, mixed with iron oxide reds, zinc white, and Barite or lithopone as fillers. These layers also have Pb, which may be related to red or white lead pigment.

The detection of Cr and Pb suggests chrome yellow, while Fe may be related to an ochre pigment in M9, mixed with white pigments and fillers.

The light blue sky in M5 was possibly obtained with cerulean blue, a cobalt stannate mixed with zinc, and lead white. This and other blue or purple-blue layers also contain aluminosilicates, which could be related to ultramarine blue or other fillers.

The dark regions in M5 (gray clouds) and M6 (brown ground) primarily contain Fe and Zn, suggesting the use of earth pigments and iron oxides mixed with zinc white and aluminosilicate fillers. Also, some carbon black was observed.

Finally, the purple layer (II) in M2 contains Si, Al, and zinc white, which are not clearly associated with inorganic purple or violet pigments. This may indicate the use of an organic pigment to achieve the purple hue. This purple layer could be related to an earlier version of the painting in which a purple skirt was depicted, as seen in a color photograph taken by Nicholas Muray in 1939 (Figure S2).

The palette used by Kahlo in *Las Dos Fridas* aligns with those employed by her contemporaries between 1930 and 1940 and with materials manufactured in the 1940s. It includes traditional pigments such as lead white and mercury sulfide, as well as more modern pigments like cadmium red and possibly organic pigments (Tables S3 and S4).

The ATR-FTIR signals at 1538, 1455, 743, and 723 cm^–1^ confirm the presence of zinc ions in M3. Their formation may explain the presence of zinc-rich particle conglomerates, likely resulting from the dissolution of acicular zinc white particles. The growth of these metal soaps could modify the painting’s light absorption properties, potentially accounting for the variations in hue observed in UV photography.^7^

The differences in morphology of zinc white could explain the differential fluorescence in UV photography between the region of the skirt, which fluoresces in a greenish hue, and the blue fluorescence of the torso.^3^ However, it is necessary to consider additional factors such as pigment–binder interactions, aging, and the presence of other compounds. The morphology of zinc white could vary the concentration of stronger O^2–^ basic sites on its surface, thereby affecting its catalytic activity.^22^

The identification of DDT with NMR in the microsamples was unexpected, as no documentation of fumigation efforts in the vicinity of the painting exists. Pesticides such as DDT have been widely employed as preventive and curative conservation treatments for cultural and biological collections, safeguarding them against pest and mold infestations. However, these chemicals pose health risks to staff members who are exposed to contaminated objects. Organochlorine pesticides, being lipophilic compounds, gradually accumulate in the body over time.^23^

The presence of zinc lactate has been reported in several papers as a degradation product associated with zinc white pigment and lactic acid coming from the environment.^5,7−10^ However, as observed in Table 1, zinc lactate is distributed in several layers across the stratigraphic sequence of the microsamples and not only in the surface layers in contact with the environment or the canvas. Another possible source of lactic acid is fungi colonizers, which can compromise plant-based materials, causing structural damage to natural fibers by making them susceptible to lignocellulosic enzymes, producing short-chain acids as a result of their metabolism.^24−26^ In this study, fungal growth was observed neither macroscopically, by visual inspection of the artwork, nor microscopically, by SEM analysis of the cross sections. Thus, the formation of lactic acid and, consequently, zinc lactate cannot be attributed to fungal metabolism.

In this work, considering the composition of *Las Dos Fridas* and the observations made so far, a metal-catalyzed radical pathway is proposed to explain the presence of zinc lactate in the work of art. This proposal is based on the catalytic synthesis of organic acids from biomass.^27^

The first step in biomass transformation involves cellulose depolymerization. The process of cleavage of the glycosidic bond of cellulose, either in aqueous solution or in a solid phase, can occur in several ways, including basic media where hydroxyl radicals break the saccharide rings and glycosidic bonds.^28,29^ The decomposition of cellulose requires an activation energy of 178 kJ mol^–1^.^30^ However, the activation energy and apparent activation energy of the oxidation of cotton fibers may decrease when soaked in linseed oil.^31^ Linseed oil contains mostly bis-allylic triglycerides that can generate free radicals, promoting the oxidation and pyrolysis of cellulose.^32−37^ During the initiation of the curing process of oil paints, bis-allyl radicals are generated, allowing linseed oil to react with oxygen and form highly reactive peroxyl radicals, which further decompose into alkoxyl and hydroxyl radicals.^38,39^ Fan et al. demonstrate the cellulose photodegradation to 5-HMF under ultraviolet light catalyzed by TiO_2_ and ZnCl_2_ via free radicals. The authors suggest the photocatalytic formation of hydroxyl radicals on the surface of TiO_2_, which can decompound the cellulose chain.^40^ A key factor in the breaking of the glycosidic bond is the presence of a molten salt hydrate as Zn^2+^ for hydrogen bond breaking, which could come from the solubilization of the zinc white pigment in the linseed oil matrix and in water nanoclusters dispersed at the pigment–polymer interface.^41,42^ It is proposed that the cleavage of the glycosidic bond in the cotton canvas is carried out by hydroxyl-free radicals generated during the autoxidation process of linseed oil.

In the palette identified in *Las Dos Fridas*, zinc white was the most distributed pigment. Generally, pure zinc oxide is considered to have no effect on the oxidation rate of linseed oil through redox reaction.^43^ However, ZnO is classified as an auxiliary drying agent, increasing the rate of oxygen uptake to the polymer layer.^44−46^ Although zinc white was identified in most layers of the microsamples, the palette also included pigments like cerulean blue, iron oxides, and lead white that can act as surface driers that catalyze the decomposition of the hydroperoxide, resulting in the formation of peroxy and alkoxy radicals.^43^

ZnO is effective in the catalytic conversion of glucose to lactic acid under hydrothermal conditions. Its catalytic activity is mainly attributed to its strong Lewis acid and base sites, which have been shown to be responsible for the desirable catalytic performance.^47^ Additionally, the crystalline defects in ZnO could catalyze the degradation of drying oils.^48^ However, *Las Dos Fridas* has not been exposed to outdoor conditions, as is the case with some contemporary murals, which present several alterations.^49^ Consequently, it does not meet the necessary conditions for catalysis under hydrothermal conditions, as reported by Xu et al. However, there are cases of the room temperature catalysis of glucose to short-chain acids. For example: (i) catalysis of glucose to formate with ZnO nanoparticles at 25 °C in the presence of NaOH 0.03 M,^50^ (ii) quantitative and semiquantitative catalysis of glucose to formic acid in anaerobic conditions catalyzed by Ba(OH)_2_,^51^ (iii) via free radicals with Li(OH),^52^ and (iv) a moderated yield of lactic acid could be obtained in solid state via mechanosynthesis at 40 °C catalyzed by Ba(OH)_2_.^53^ In general terms, the reported mechanism in the catalysis of glucose involves the formation of fructose or xylose, followed by a retro-aldol reaction in a basic medium to obtain C3 species, such as glyceraldehyde and dihydroxyacetone. Finally, short-chain acids, such as formic, lactic, or glycolic acids, can be formed by water loss, mainly through a radical, which can be generated on the surface of metal oxides and may persist for several days.^54,55^

This research proposes that ZnO could act as a source of Lewis acids and bases, catalyzing the formation of lactic acid. The lack of a consistent preparation layer promoted the oxidation of the cellulose of the canvas during the drying process of linseed oil, which probably occurred since the manufacture of *Las Dos Fridas*.

## Conclusions

The spectroscopic and microscopic analysis of *Las Dos Fridas* reveals a complex interplay between the materials used by Kahlo and the occurrence of several chemical degradation processes over a long time. The unexpected identification of DDT points to undocumented fumigations near the painting, raising concerns about the possible interactions with the materials of the painting and potential health risks posed by pesticide residues. The detection of zinc lactate, distributed throughout several layers of the painting rather than just on the surface, suggests a degradation pathway associated with the zinc white pigment. This study proposes that a metal-catalyzed radical pathway, likely involving zinc oxide from the pigment, may have catalyzed the oxidation of cellulose in the canvas, leading to the formation of lactic acid. This degradation process, driven by free radicals from the drying of the linseed oil, offers new insights into the chemical changes that affected the structural integrity of *Las Dos Fridas* and provides a deeper understanding of the long-term conservation challenges for artworks of this type. In the proposed mechanism, the presence of water influences both the catalytic processes and the formation of Zn^2+^ ions. Therefore, it is recommended that cleaning methods that involve the use of water be avoided. Further research is necessary to understand the evolution of this kind of degradation as well as to determine the presence of zinc lactate by means of imaging spectroscopic techniques.

## Supporting Information Available

Identification of cotton fibers in Las Dos Fridas by modified Herzog test (Figure S1); color photograph of Kahlo painting her double portrait by Nicholas Muray (Figure S2); spectroscopic data δ_H_, δ_C_ HSQC, HMBC, and COSY (300 K, CDCl_3_) from linseed oil and DDT (Figures S3 and S4, Tables S1 and S2); SEM micrograph (BSE, 15.0 kV) and EDS elemental maps of microsamples prepared as cross sections of *Las Dos Fridas* (Figures S5–S10); materials used by Kahlo contemporary artist and materials manufactured in 1940s (Tables S3 and S4) (PDF)

## Footnotes

1 Zetina, S. Reporte del estudio material de Las Dos Fridas, Frida Kahlo, 1939. (2008). Laboratorio de Diagnóstico de Obras de Arte, del Instituto de Investigaciones Estéticas de la Universidad Nacional Autónoma de México (The Art Diagnostic Laboratory of the Aesthetic Research Institute from National Autonomous University of Mexico, LDOA-IIE-UNAM).
