# Supplementary material for: Characterization by Spectroscopic and Microscopic Techniques of Degraded Zinc White Pigment in Las Dos Fridas
Source: ACS Omega. 2025 Apr 18;10(16):16309–18. doi: 10.1021/acsomega.4c10530 (PMC12044470; doi:10.1021/acsomega.4c10530)
Supplement: Supplementary file 1 — ao4c10530_si_001.pdf [file ao4c10530_si_001.pdf]

Supplementary Information: Characterization by Spectroscopic and Microscopic Techniques of Degraded Zinc White Pigment in *Las Dos Fridas*

Pablo Aguilar-Rodríguez[a], Sandra Zetina[b], Adrián Mejía-González[a], and Nuria Esturau-Escofet*[a]

[a] Instituto de Química, Universidad Nacional Autónoma de México, México City 04510, México; [b] Instituto de Investigaciones Estéticas, Universidad Nacional Autónoma de México, México City 04510, México.
*Corresponding author, email: nesturau@iquimica.unam.mx

Table of Contents

Figure S1 Identification of cotton fibers in Las Dos Fridas by modified Herzog test. a) Polarized longitudinal section. Sample orientation angle b) α = 0° and c) α = 90°. 2
Figure S2. Nicholas Muray, Frida Kahlo painting, circa September 1939, color transparency, Kodachrome development, 24 x 36 mm, George Eastman House. 2
Figure S3. ed-HSQC (red - blue) and HMBC (green) spectra (700 MHz, 300 K, $CDCl_3$) of microsample M5 of Las Dos Fridas. The structure of the palmitic and oleic triglyceride with the key correlation and the assignment of the signals in the spectra are shown. 3
Figure S4. HSQC (red) and HMBC (green) spectra (700 MHz, $CDCl_3$) of microsample M5. The structure of the DDT with the key correlation and the assignment of the signals in the spectra are shown. 4
Figure S5. a) SEM micrograph (BSE, 15.0 kV) of cross section from sample M2 of Las Dos Fridas and EDS elemental maps: b) O, c) Zn, d) Pb, e) Cr, f) Hg, g) Si, h) Al and i) Mg. 5
Figure S6 a) SEM micrograph (BSE, 15.0 kV) of cross section from sample M3 of *Las Dos Fridas* and EDS elemental maps: b) O, c) Zn, d) Si, e) Cl and f) S. 6
Figure S7. a) SEM micrograph (BSE, 20.0 kV) of cross section from sample M5 of Las Dos Fridas and EDS elemental maps: b) O, c) Zn, d) Co, e) Sn, f) Pb, g) Fe, h) Al, i) Si, j) Mg, k) Ca, l) K and m) Cl. 7
Figure S8 a) SEM micrograph (BSE, 20.0 kV) of cross section from sample M6 of Las Dos Fridas and EDS elemental maps: b) O, c) Zn, d) Si, e) Ca, f) Fe and g) Al. 8
Figure S9. a) SEM micrograph (BSE, 15.0 kV) of cross section from sample M8 of Las Dos Fridas and EDS elemental maps: b) O, c) Zn, d) S, e) Cd, f) Se, g) Al, h) Si, i) Mg, j) Fe and k) Ca. 9
Figure S10. a) SEM micrograph (BSE, 20.0 kV) of cross section from sample M9 of Las Dos Fridas and EDS elemental maps: b) O, c) Zn, d) Cr, e) Pb, f) Fe, g) Cd, h) Se, i) Al, j) Si and k) Cl. 10

Table S1. Spectroscopic data: δ_H, δ_c, HMBC and COSY (300 K, $CDCl_3$) of palmitic and oleic triglyceride identified in Las Dos Fridas. 3
Table S2. Spectroscopic data δ_H, δ_c, HMBC and COSY (300 K, $CDCl_3$) of DDT identified in Las Dos Fridas. 4
Table S3 Palette of oil painting pigments used by contemporary artists during the period of Las Dos Fridas. 11
Table S4 Composition of oil paint samples from historical collapsible tubes manufactured by HKS, materials contemporary with the period of *Las Dos Fridas*.[5] 11

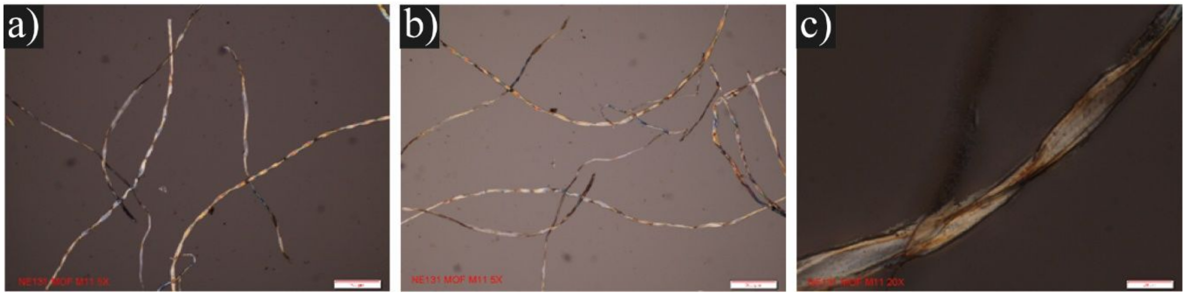

Figure S1 Identification of cotton fibers in *Las Dos Fridas* by modified Herzog test. a) Polarized longitudinal section. Sample orientation angles: b) $\alpha = 0°$ and c) $\alpha = 90°$.

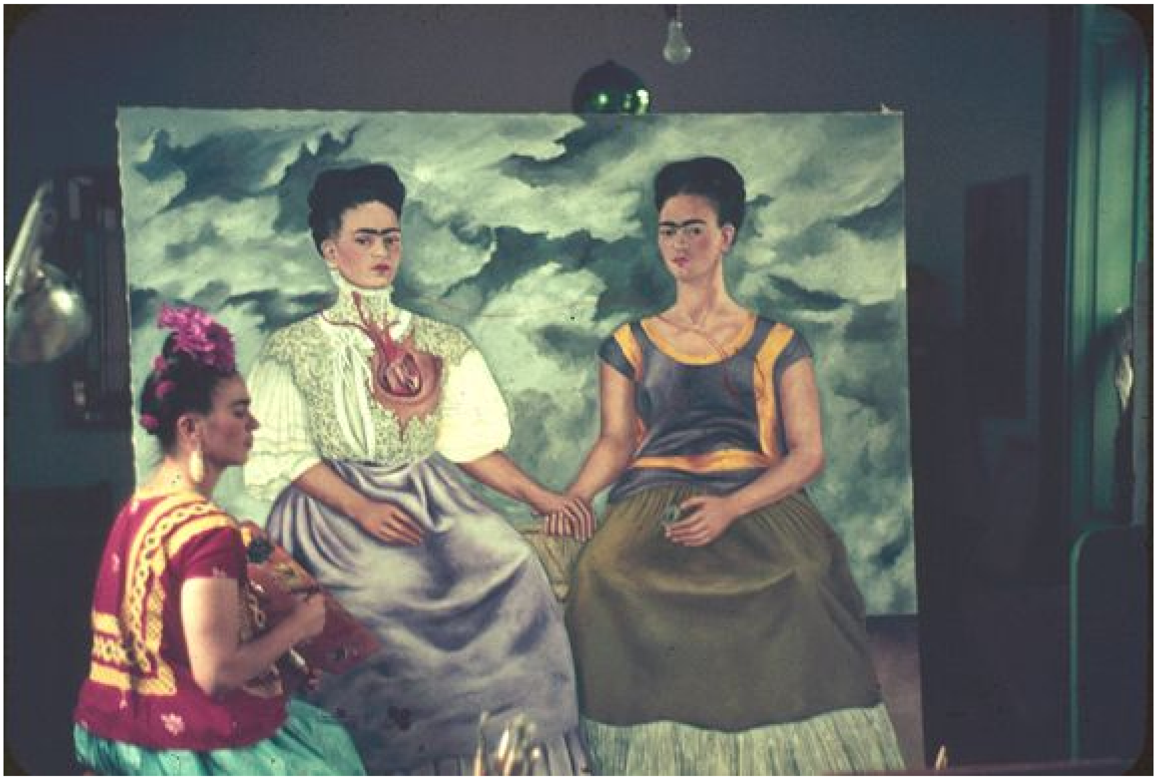

Figure S2. *Nickolas Muray, Frida Kahlo with her painting, The Two Fridas, 1939, chromogenic development transparency, (2.3 × 3.3 cm), George Eastman House, Rochester New York. All rights reserved: copyright 2024 Banco de México, Fiduciario en el Fideicomiso relativo a los Museos Diego Rivera y Frida Kahlo. Av. 5 de Mayo No. 2, col. Centro, alc. Cuauhtémoc, c.p. 06000, Ciudad de México. Reproducción autorizada por el INBAL*

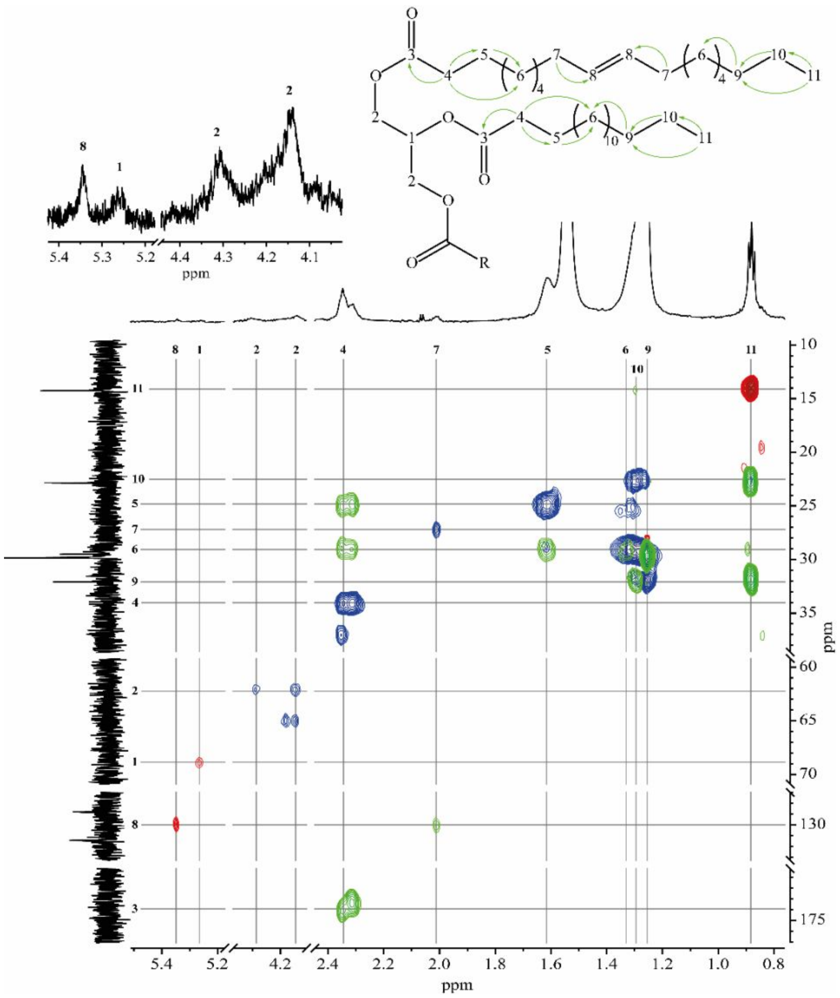

Figure S3. ed-HSQC (red - blue) and HMBC (green) spectra (700 MHz, 300 K, $CDCl_3$) of microsample M5 of *Las Dos Fridas*. The structure of the palmitic and oleic triglyceride with the key correlation and the assignment of the signals in the spectra are shown.

Table S1. Spectroscopic data: δ_H, δ_c, HMBC and COSY (300 K, $CDCl_3$) of palmitic and oleic triglyceride identified in *Las Dos Fridas*.

**Label**	**$\delta_{H\,/}$ ppm**	**$\delta_{C\,/}$ ppm**	**HMBC (H→C)**	**COSY (H→H)**
**1**	5.26	68.9		
**2**	4.29 and 4.15	62.2		
**3**		174.0		
**4**	2.34	37.2	C – 3, 5, 6	H – 5
**5**	1.62	24.8	C – 6	H – 4
**6**	1.33	29.1	C – 6	
**7**	2.01	27.2	C – 8	
**8**	5.35	130.0		
**9**	1.29	32.1	C – 6	H – 10
**10**	1.25	29.7	C – 9	H – 9, 11
**11**	0.88	14.1	C – 9, 10	H – 10

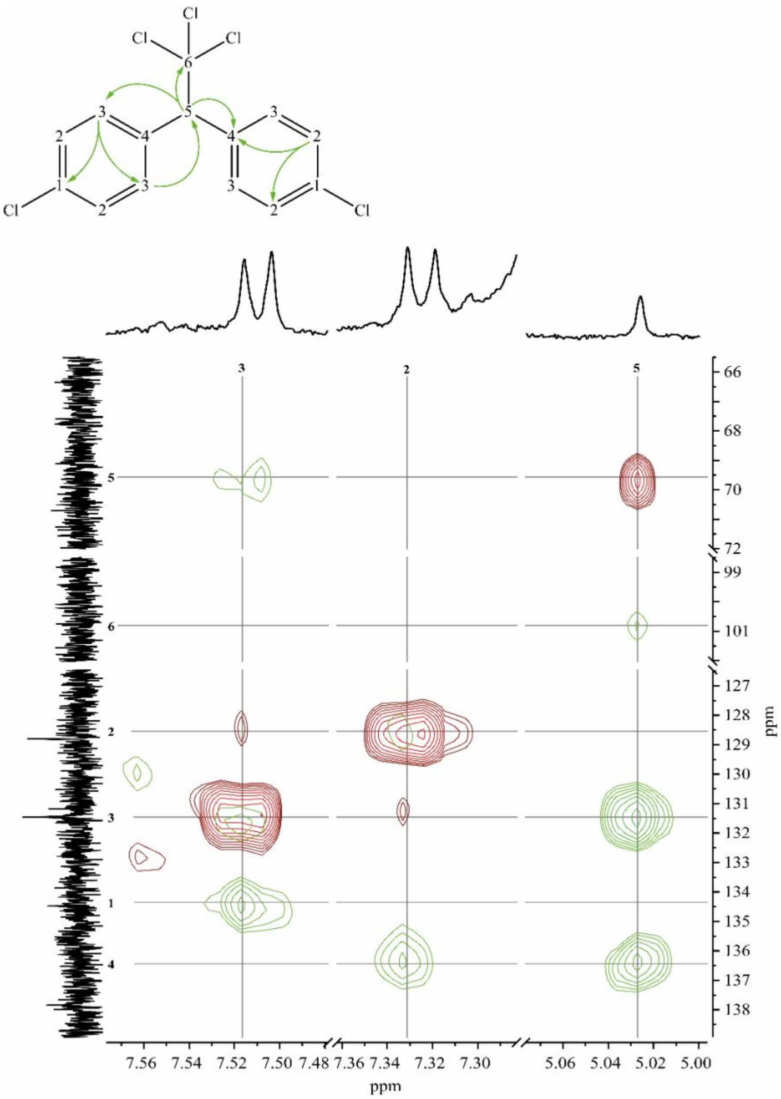

Figure S4. HSQC (red) and HMBC (green) spectra (700 MHz, $CDCl_3$) of M5. The structure of the DDT with the key correlation and the assignment of the signals in the spectra are shown.

Table S2. Spectroscopic data δ_H, δ_c, HMBC and COSY (300 K, $CDCl_3$) of DDT identified in *Las Dos Fridas*.

**Label**	**δ_{H} / ppm** (multiplicity, **J/ Hz)**	**δ_{C} / ppm**	**HMBC (H→C)**	**COSY (H→H)**
**1**	-	134.5	-	-
**2**	7.32 (d, 8.6)	128.7	C-2, 4	H-3
**3**	7.51 (d, 8.4)	131.4	C-1, 3, 5	H-2
**4**	-	136.3	-	-
**5**	5.02 8 (s)	69.7	C-3, 4, 6	-
**6**	-	100.8	-	-

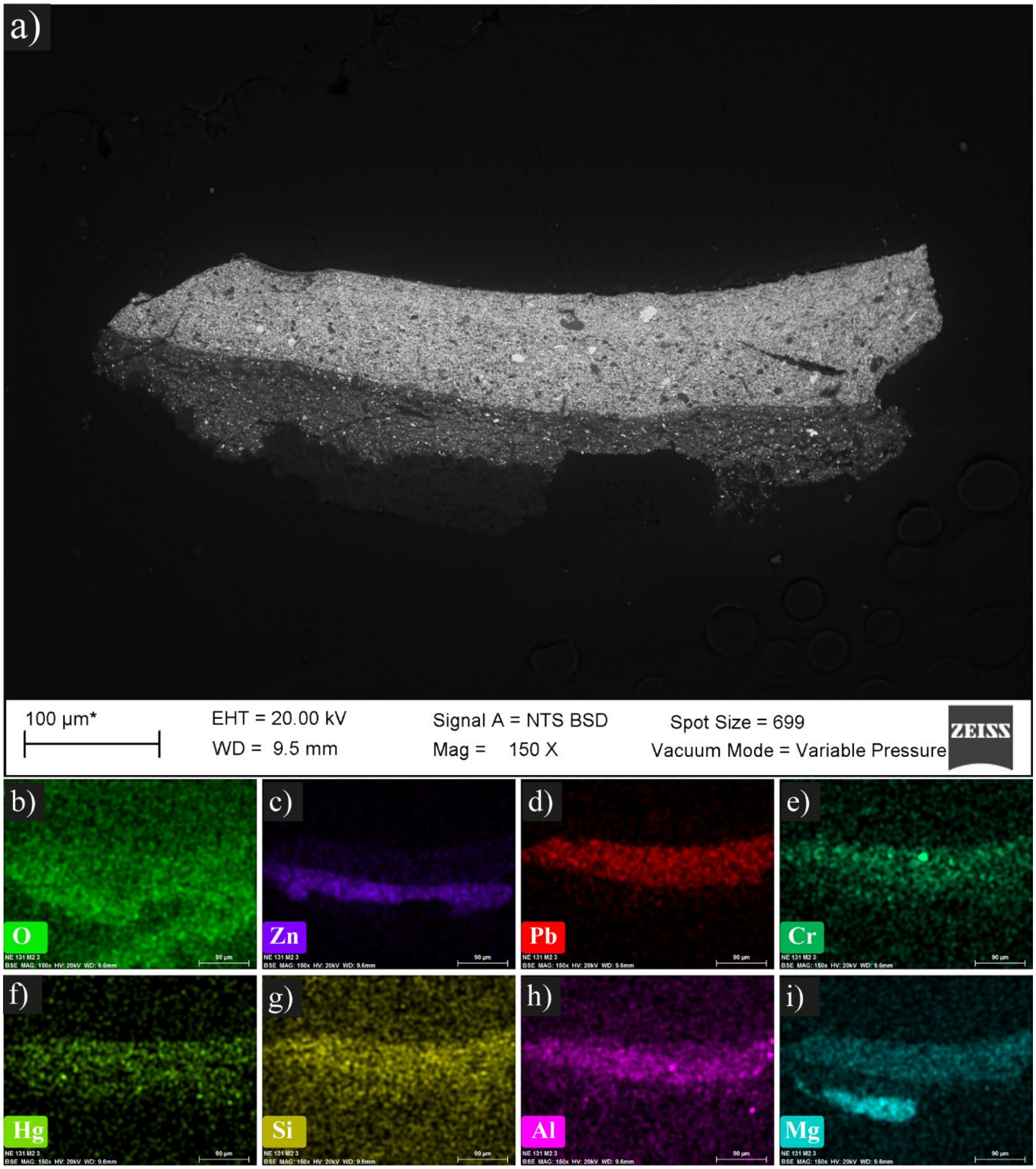

Figure S5. a) SEM micrograph (BSE, 15.0 kV) of cross section from M2 of Las Dos Fridas and EDS elemental maps: b) O, c) Zn, d) Pb, e) Cr, f) Hg, g) Si, h) Al and i) Mg.

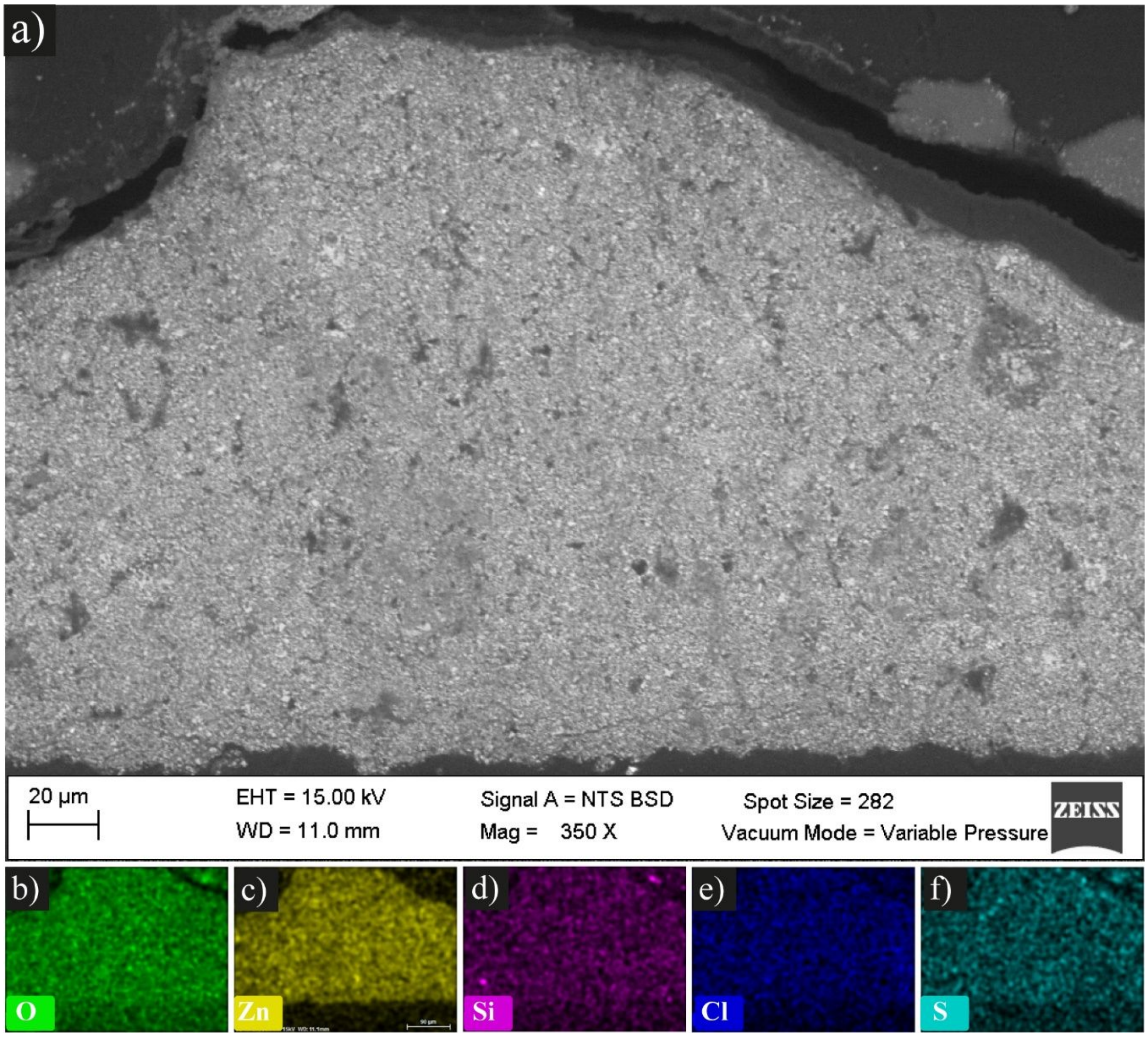

Figure S6 a) SEM micrograph (BSE, 15.0 kV) of cross section M3 of *Las Dos Fridas* and EDS elemental maps: b) O, c) Zn, d) Si, e) Cl and f) S.

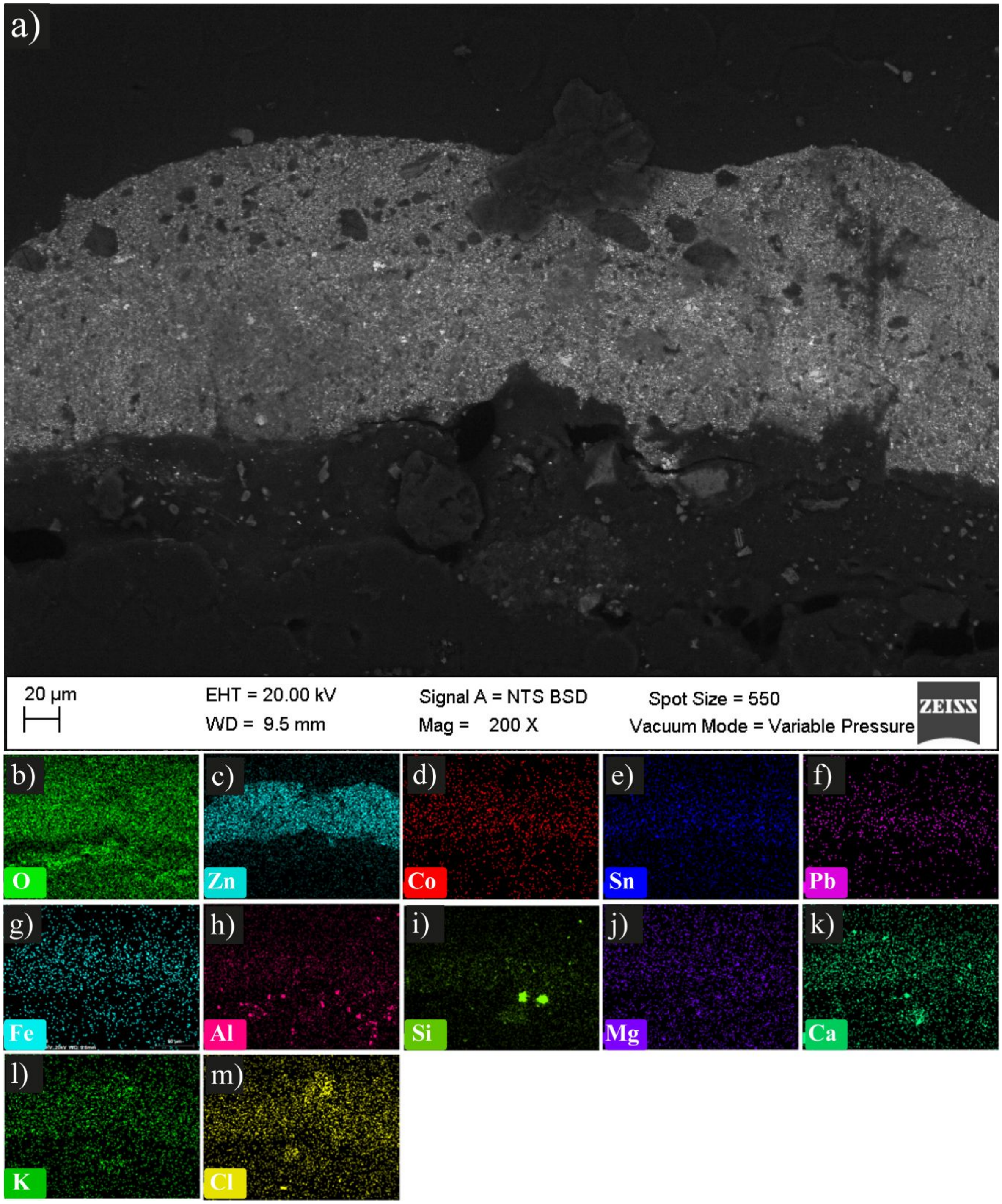

Figure S7. a) SEM micrograph (BSE, 20.0 kV) of cross section from M5 of *Las Dos Fridas* and EDS elemental maps: b) O, c) Zn, d) Co, e) Sn, f) Pb, g) Fe, h) Al, i) Si, j) Mg, k) Ca, l) K and m) Cl.

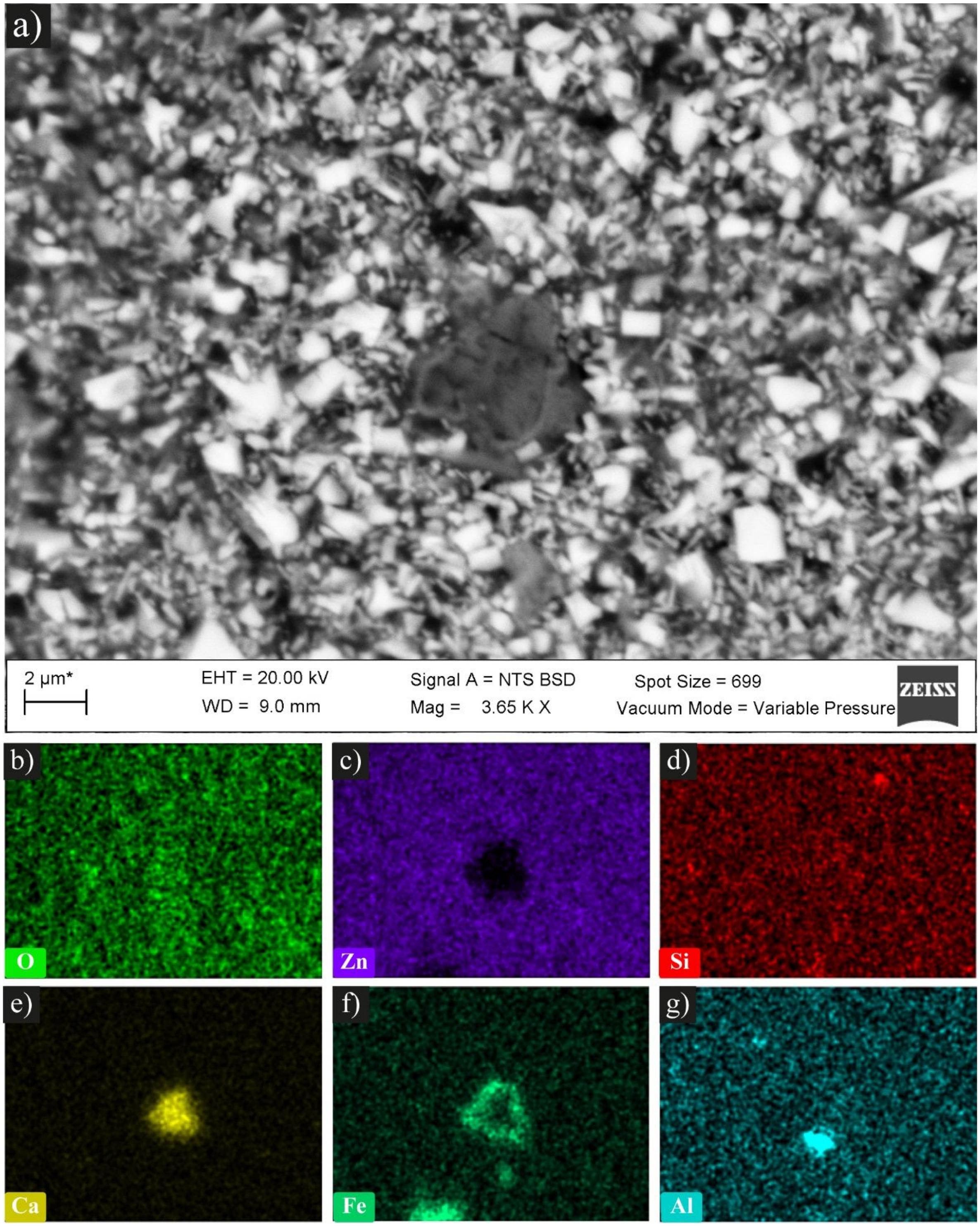

Figure S8 a) SEM micrograph (BSE, 20.0 kV) of cross section from M6 of *Las Dos Fridas* and EDS elemental maps: b) O, c) Zn, d) Si, e) Ca, f) Fe and g) Al.

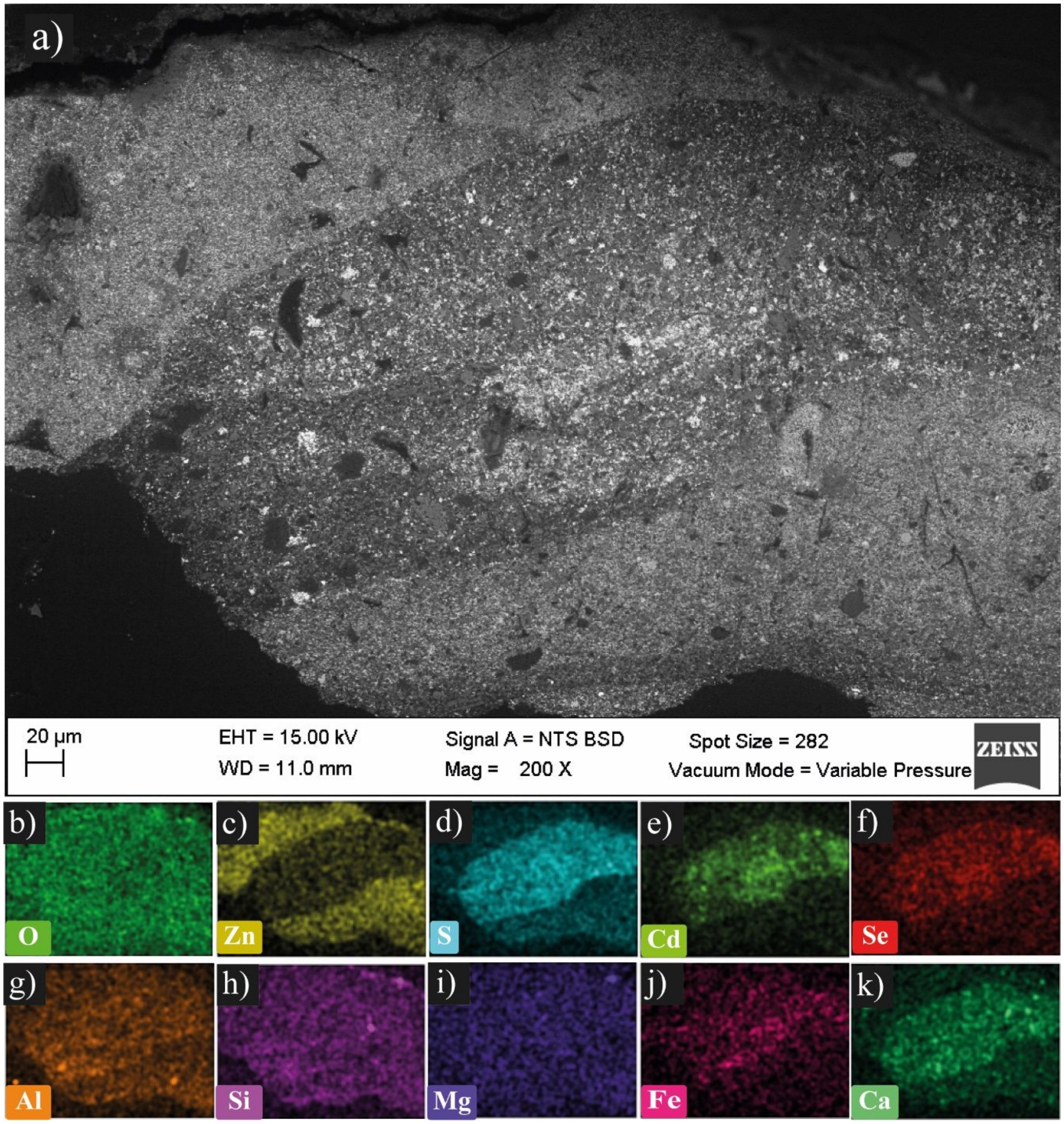

Figure S9. a) SEM micrograph (BSE, 15.0 kV) of cross section from M8 of *Las Dos Fridas* and EDS elemental maps: b) O, c) Zn, d) S, e) Cd, f) Se, g) Al, h) Si, i) Mg, j) Fe and k) Ca.

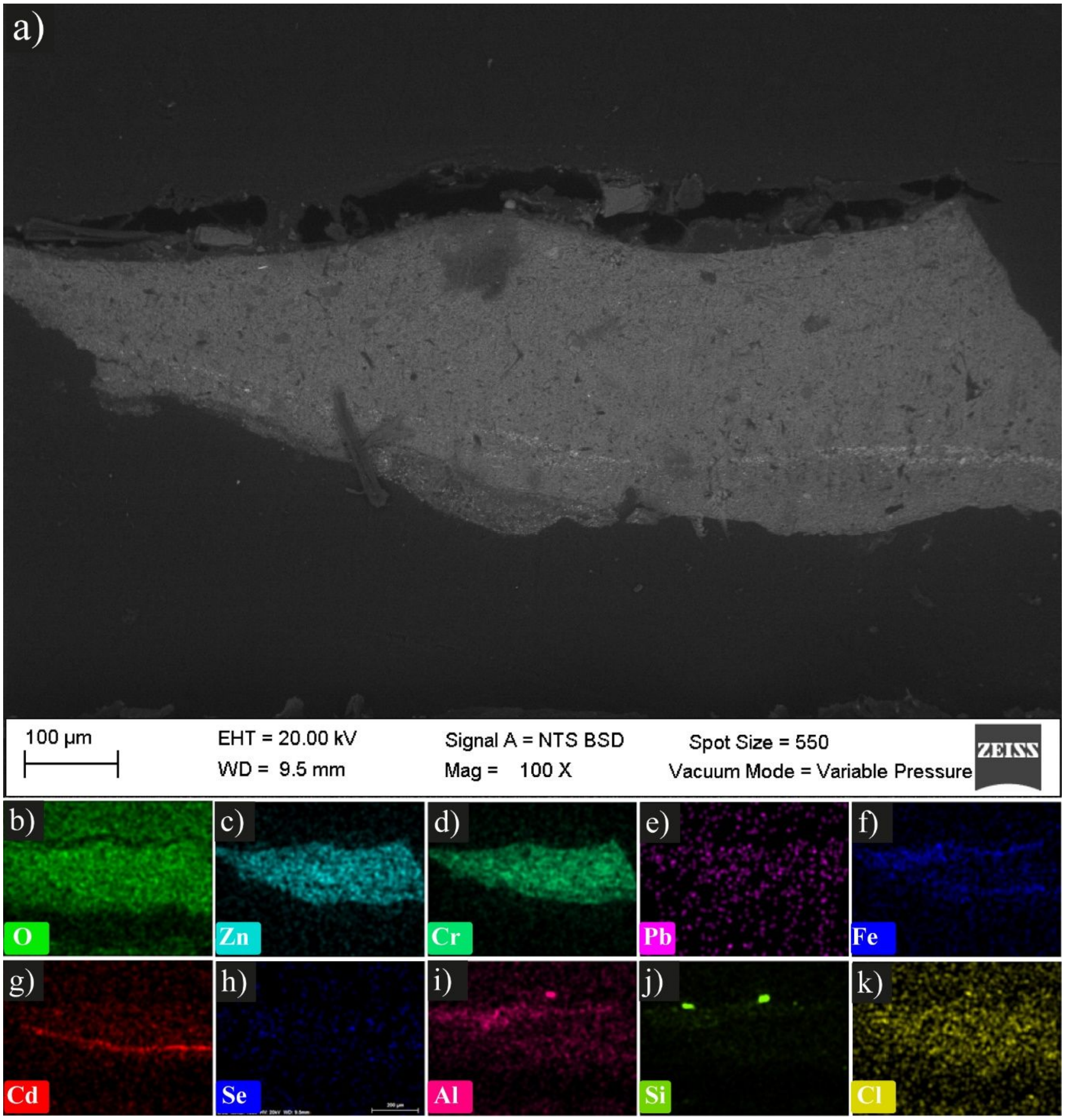

Figure S10. a) SEM micrograph (BSE, 20.0 kV) of cross section from M9 of *Las Dos Fridas* and EDS elemental maps: b) O, c) Zn, d) Cr, e) Pb, f) Fe, g) Cd, h) Se, i) Al, j) Si and k) Cl.

Table S3 Palette of oil painting pigments used by contemporary artists during the period of *Las Dos Fridas*.

Year	Artist	Artwork	Reported palette	Reference
1935	Borlase Smart	Seascape at Clodgy	Cobalt blue, lead (zinc) chromate lead white, red lake, viridian, zinc oxalate	[1]
1936	Salvador Dalí	Couple with Clouds	Cadmium yellow, cerulean blue, cobalt violet, zinc white	[2]
1938	Josef Albers	Slanting Cross	Calcium carbonate, cobalt blue, Cu-arsenate green, viridian, zinc white	[3]
1938-1944	Josef Albers	Related I	Barium sulfate, Cd-based reds, kaolin, organic red, zinc white	[3]
1942	Guido Cadorin	Ritratto di Giovanni	Barium white/lithopone, burn umber, carbon black, chalk, gypsum, hematite, zinc white	[4]
1943	Guido Cadorin	La Navicella di San Pietro	Carbon black, cinnabar/vermilion, lead white, titanium white, ultramarine blue, zinc white	[4]
1943	Josef Albers	Variant of Related	Barium sulfate, cadmium red, cobalt blue, kaolin, organic red, ultramarine blue, zinc white	[3]
1944	Josef Albers	Tautonym B	Barium sulfate, bone black, Cd-based red, kaolin, phthalocyanine blue, viridian	[3]

Table S4 Composition of oil paint samples from historical collapsible tubes manufactured by HKS, materials contemporary with the period of *Las Dos Fridas*. [5]

Production Period	Brand	Paint labels	Reported compounds
c. 1940	HKS	Goud Oker	Aluminum stearate, barium sulfate, calcium carbonate, Iron oxide, kaolin
		Kobalt Violet	Aluminum stearate, calcium carbonate, cobalt violet
		Monastraal Blauw	Aluminum stearates, chromium oxide, gypsum, iron oxide, phthalocyanine blue, titanium white
		ChromeGreen	Aluminum stearate, beeswax, barium sulphate, calcium carbonate, chromium oxide
		EmeraldGreen	Aluminum stearate, gypsum, phthalocyanine blue

REFERENCES

(1) Burntock, A.; van den Berg, J.; de Groot, S.; Wijnberg, L. An Investigation of Water-Sensitive Oil Paints in 20th Century Paintings. In *Modern Paints Uncovered: Proceedings from the Modern Paints Uncovered Symposium*; Learner, T. J. S., Smithen, P., Krueger, J. W., Schilling, M. R., Eds.; Getty Conservation Institute: Los Angeles, 2007; pp 177–188.

(2) Keune, K.; Boevé-Jones, G. Its Surreal: Zinc-Oxide Degradation and Misperceptions in Salvador Dalí's Couple with Clouds in Their Heads, 1936. In *Issues in Contemporary Oil Paint*; Springer International Publishing: Cham, 2014; pp 283–294. https://doi.org/10.1007/978-3-319-10100-2_19.

(3) Poldi, G.; Anselmi, C.; Daveri, A.; Vagnini, M. CHAPTER 4. Josef Albers' Use of 20th Century Pigments: A Non-Invasive Analytical Approach. In *Science and Art*; Royal Society of Chemistry: Cambridge, 2020; pp 67–94. https://doi.org/10.1039/9781788016384-00067.

(4) Morales Toledo, E. G.; Raicu, T.; Falchi, L.; Barisoni, E.; Piccolo, M.; Izzo, F. C. Critical Analysis of the Materials Used by the Venetian Artist Guido Cadorin (1892–1976) during the Mid-20th Century, Using a Multi-Analytical Approach. *Heritage* 2023, *6* (1), 600–627. https://doi.org/10.3390/heritage6010032.

(5) Izzo, F. C.; van den Berg, K. J.; van Keulen, H.; Ferriani, B.; Zendri, E. Modern Oil Paints – Formulations, Organic Additives and Degradation: Some Case Studies. In *Issues in Contemporary Oil Paint*; Springer International Publishing: Cham, 2014; pp 75–104. https://doi.org/10.1007/978-3-319-10100-2_5.
